# Clinical Characteristics, Etiologies, Management, and Outcomes of Adult Exfoliative Dermatitis at Chao Phraya Abhaibhubejhr Hospital, Thailand: A Prospective Cohort Study

**DOI:** 10.7759/cureus.105256

**Published:** 2026-03-15

**Authors:** Pitchaya Jaruvijitrattana, Weeratian Tawanwongsri

**Affiliations:** 1 Division of Dermatology, Department of Internal Medicine, Chao Phraya Abhaibhubejhr Hospital, Prachinburi, THA; 2 Division of Dermatology, Department of Internal Medicine, School of Medicine, Walailak University, Nakhon Si Thammarat, THA

**Keywords:** cohort studies, dermatitis, drug hypersensitivity, erythroderma, exfoliative, glucocorticoids, psoriasis

## Abstract

Introduction

Adult exfoliative dermatitis (ED) is a severe inflammatory dermatosis with heterogeneous etiologies and substantial morbidity. Hospital-based prospective data remain limited in Thailand. We aimed to prospectively describe the clinical characteristics, etiologies, management, and outcomes of adults with ED at a tertiary-care hospital in Thailand, and to assess changes during follow-up.

Methods

We conducted a prospective hospital-based cohort study enrolling consecutive adults diagnosed with ED from November 2024 to March 2026. Baseline demographics, comorbidities, prior treatments, clinical manifestations, and disease severity were recorded. Etiology was determined by clinicopathologic correlation and treating-physician adjudication. Patients were followed longitudinally for up to 12 months after diagnosis, and changes in disease severity and clinical outcomes were assessed during follow-up. Analyses were primarily descriptive. Exploratory comparisons across follow-up visits were reported using nonparametric tests with corresponding p-values.

Results

Thirty-four patients were recruited; two were lost to follow-up, and one had incomplete baseline data, leaving 31 for analysis. The mean age was 58.0 ± 18.2 years, and 74.2% were male. Most were managed as outpatients (67.7%), while 32.3% required hospitalization. Pruritus, scaling, and erythema were present in all patients. Nail involvement was frequent (64.5%), whereas pustules and mucosal involvement were uncommon (6.5% each). Systemic features occurred in a subset, including arthritis (25.8%), fever (16.1%), edema (16.1%), and lymphadenopathy (6.5%).

Conclusions

In this prospective hospital-based cohort, adult ED demonstrated uniform core cutaneous features with variable systemic involvement and a substantial proportion requiring hospitalization. These findings provide pragmatic clinical profiles to support early recognition and structured evaluation of etiology and complications in routine practice.

## Introduction

Exfoliative dermatitis (ED), also referred to as erythroderma, is a rare inflammatory skin disease characterized by the presence of a generalized erythema and desquamation covering more than 90% of the body surface area, often associated with pruritus, edema, and systemic manifestations [1, 2]. The reported incidence and causes vary in different parts of the world. In Kenya, ED constituted approximately 13.0% of dermatological admissions, with dermatoses and HIV as the predominant etiological factors; the hospital mortality rate was approximately 10.0% [3]. In Nigeria, the outpatient prevalence was 2.7%, with 70.0% of young adults being HIV-positive, while 50.0% of older adults had a suspected malignant condition [4]. In an extensive Chinese study, the most frequent cause of ED was a pre-existing dermatosis (70.8%), followed by idiopathic causes (14.2%), drug-induced (12.7%), and malignant disease (2.3%). Psoriasis was the most frequent dermatosis, and carbamazepine was the most common drug cited as the offending agent [5]. In Iran, psoriasis (40.7%) is also the predominant cause, followed by drug reactions and lymphoma (each 18.6%), in which co-trimoxazole (27.3%) and carbamazepine (18.2%) were the drugs involved [6]. Mistry et al. found that contact dermatitis, atopic dermatitis, psoriasis, and hypersensitivity to drugs were the principal causes, while the most common malignancy was cutaneous T-cell lymphoma. Various reports have stated that 9.0%-47.0% of cases lacked a definitive cause [7]. In a prospective Brazilian report on 309 cases, eczema (20.7%) and psoriasis (16.8%) were the most frequent causes, followed by Sézary's syndrome and drug eruptions (each 12.3%), with histopathology confirming the diagnosis in 72.4% of the patients [8]. In Thailand, a hospital-in-ward study reported an incidence of 160.0 per 100,000 dermatological outpatients per annum (approximately 32 cases per annum), and the most frequent causes were psoriasis (53.1%) and eczema (31.3%) [9].

Diagnostic confirmation typically includes combinations of clinical presentation, targeted laboratory investigation, and histology; advanced testing (Sézary cells, flow cytometry, and T-cell receptor studies) is only pursued if there is concern for cutaneous T-cell lymphoma [10-14]. Etiologic patterns fall broadly into four categories: pre-existing dermatoses (contact dermatitis, atopic dermatitis, psoriasis, pityriasis rubra pilaris, and seborrheic dermatitis); autoimmune/inflammatory diseases (bullous pemphigoid, pemphigus foliaceus, systemic lupus erythematosus, dermatomyositis, lichen planus, and graft-versus-host disease); infections/infestations and genodermatoses (HIV disease, dermatophytosis, Norwegian scabies, and ichthyosis); and drug eruptions or hematologic malignancies (cutaneous T-cell lymphoma and Sézary syndrome plus other lymphoma/leukemia) [7]. A synthesis of cross-regional summaries suggests that preexisting dermatoses are the leading cause (reported 27%-72%); drug-induced disease is typically the second cause (5%-39%), malignancy cases are often the least frequent (3%-12%), and cases classified as idiopathic range from 6%-16% [15]. This is consistent with large series from China, Brazil, and Iran [5, 6, 8].

However, prospective evidence in Thailand remains limited. Locally generated data are needed to strengthen diagnostic pathways, optimize management strategies, and inform referral systems. Therefore, this prospective hospital-based cohort study aimed to characterize the clinical features, etiologies, management, and outcomes of adults with ED at a tertiary-care hospital in Thailand and to assess longitudinal changes in disease severity and clinical outcomes during follow-up.

## Materials and methods

Study design and setting

This prospective hospital-based cohort study was conducted at Chao Phraya Abhaibhubejhr Hospital, a tertiary care center in Prachinburi Province, Thailand, between November 2024 and March 2026 (a 16-month enrollment period). Participants were followed for up to 12 months after diagnosis with prespecified assessments at months three, six, nine, and 12. Adults aged ≥18 years with ED, defined as generalized erythema and scaling involving >90% of the body surface area, were consecutively enrolled from dermatology outpatient and inpatient services. The diagnosis was established clinically by a board-certified dermatologist on the basis of diffuse erythema and scaling with estimated involvement of more than 90% of the total body surface area during routine dermatologic examination. Participants were excluded if they declined participation or withdrew consent. Cases with incomplete baseline data or without post-enrollment confirmation of the diagnosis were excluded from the analytic dataset. The study was approved by the Ethics Committee of Chao Phraya Abhaibhubejhr Hospital (IRB-BHUBEJHR-339) and conducted in accordance with the Declaration of Helsinki and International Council for Harmonization Good Clinical Practice. Written informed consent was obtained from all participants.

Data collection, outcome measures, and follow-up

Clinical data were collected from all participants using a standard case record form. Collected variables included age, sex, underlying diseases, clinical presentation, extent of cutaneous involvement, laboratory investigations, histopathology, etiologic diagnosis, treatment, complications, and outcomes. The extent of cutaneous involvement was recorded as the percentage of body surface area clinically evaluated by the treating dermatologist at baseline and at three, six, nine, and 12 months. Etiologic classification depended on clinical history, medication exposure, physical examination, laboratory results, and histopathological findings when available, utilizing clinicopathologic correlation and treating-physician adjudication. Laboratory investigations included complete blood count, liver and kidney function tests, serum electrolytes, and other serologic tests as clinically indicated. A skin biopsy was performed when the diagnosis or underlying cause remained unclear after the initial clinical evaluation. Treatment was not protocolized; the treating dermatologist selected therapy according to the suspected etiology, disease severity, comorbidities, and clinical judgment. The primary objective was to describe the clinical characteristics, etiologies, management, and outcomes of adults with ED. The secondary objective was to assess longitudinal changes in disease severity and clinical outcomes during follow-up. The primary outcome was the descriptive characterization of adult ED, including clinical characteristics and etiologic distribution among consecutively enrolled patients. All patients were followed up at three, six, nine, and 12 months after diagnosis to assess disease progression, relapse, treatment-related sequelae, and overall outcomes. The global clinical outcome at each follow-up point was recorded as a four-level ordinal scale: 1 = cured, 2 = improved, 3 = unchanged, and 4 = worsened, at three, six, nine, and 12 months.

Statistical analysis

The study was designed to provide prospective descriptive evidence on adult ED, including clinical characteristics, etiologies, management, and outcomes. Sample size planning was informed by a previous Thai hospital report indicating approximately 32 ED cases annually [9]. Under a Poisson framework for rare conditions, the relative standard error (RSE) of a case count is inversely proportional to the square root of the number of observed cases. With a target of approximately 31 cases, the expected RSE was about 18%, corresponding to an approximate two-sided 95% confidence interval with a relative half-width of 35% for the underlying event rate. In the final dataset, 31 analyzable cases were included, and exact Poisson 95% confidence intervals were prespecified for the observed number of cases.

Descriptive statistics summarized participant characteristics, causes, clinical features, laboratory findings, treatments, and results. Categorical variables appear as counts and percentages. Continuous variables are shown as mean (standard deviation) or median (interquartile range), depending on what is appropriate. We analyzed longitudinal changes in body surface area at 0, three, six, nine, and 12 months using the Friedman test. When overall differences were detected, pairwise comparisons between visits were performed using Wilcoxon signed-rank tests with Bonferroni adjustment. Global clinical outcomes at three, six, nine, and 12 months were summarized as counts and percentages. For ordinal longitudinal testing, outcome categories were analyzed using the prespecified clinical order: worsened, unchanged, improved, and cured; numeric coding was reversed as needed to reflect this order. This analysis used the Friedman test, followed by Wilcoxon signed-rank tests with Bonferroni correction for post-hoc pairwise comparisons. Longitudinal analyses were restricted to patients with complete data at all relevant follow-up visits. All tests were two-sided, and statistical significance was considered at p < 0.05. Analyses were conducted using R (version 4.3.2; R Foundation for Statistical Computing, Vienna, Austria).

## Results

A total of 34 participants with adult ED were recruited; two were lost to follow-up, and one had incomplete baseline data; thus, 31 patients were included in the analysis. The baseline characteristics are reported in Table 1. The mean age was 58.0 ± 18.2 years, and 23 of 31 patients (74.2%) were male. The median BMI was 22.3 kg/m² (IQR 6.2). Most patients were managed in the outpatient setting (21/31, 67.7%), whereas 10 (32.3%) required hospitalization. Among the 31 patients included in the analysis, 20 (64.5%) had at least one chronic comorbidity, such as hypertension, psoriasis, diabetes mellitus, dyslipidemia, or chronic kidney disease. The median time from symptom onset to presentation was 60 days (IQR 45 days). Prior treatment was common, most frequently topical corticosteroids (74.2%) and oral antihistamines (16.1%). The core presentation was uniform, with pruritus, scaling, and erythema each observed in all patients. Nail involvement was frequent (64.5%), whereas pustules (6.5%) and mucosal involvement (conjunctivitis 6.5%; oral mucositis 6.5%) were uncommon. Systemic or associated features occurred in a subset, including arthritis (25.8%), fever (16.1%), edema (16.1%), and lymphadenopathy (6.5%) (Table 1).

**Table 1:** Patients’ characteristics (n = 31) Abbreviations: BSA, body surface area; BMI, body mass index; CKD, chronic kidney disease; CM, centimeter; DM, diabetes mellitus; DLP, dyslipidemia; HIV, human immunodeficiency virus; HT, hypertension; IPD, inpatient department; IQR, interquartile range; n, number; SD, standard deviation; kg, kilogram; cm, centimeter; OPD, outpatient department; TB, tuberculosis; CHF, congestive heart failure. Other systemic diseases: gout (n = 2), epilepsy (n = 1), tuberculosis of the spine (n = 1), congestive heart failure (n = 1), chronic ulcer with generalized pustules (n = 1). Data are presented as n (%) for categorical variables and as mean ± SD or median IQR, as appropriate.

Characteristics	Overall (n = 31)
Age (years), mean ± SD	58.0 ± 18.2
Male, n (%)	23 (74.2)
Weight (kg), mean ± SD	60.6 ± 12.4
Height (cm), mean ± SD	162.1 ± 11.6
BMI (kg/m²), median (IQR)	22.3 (6.2)
Visit type	
OPD, n (%)	21 (67.7)
IPD, n (%)	10 (32.3)
Comorbidities, n (%)	20 (64.5)
Hypertension, n (%)	12 (38.7)
Skin disease (psoriasis), n (%)	11 (35.5)
Diabetes mellitus, n (%)	6 (19.4)
Dyslipidemia, n (%)	6 (19.4)
Chronic kidney disease, n (%)	6 (19.4)
Other systemic diseases, n (%)	6 (19.4)
Onset (days), median (IQR)	60 (45)
Initial BSA (%), median (IQR)	100 (5)
Prior treatment, n (%)	26 (83.9)
Topical steroid, n (%)	23 (74.2)
Oral antihistamine, n (%)	5 (16.1)
Thai traditional medicine, n (%)	3 (9.7)
Other herbal product, n (%)	1 (3.2)
Oral antibiotics, n (%)	1 (3.2)
Symptoms	
Pruritus, n (%)	31 (100.0)
Pain, n (%)	16 (51.6)
Burning, n (%)	14 (45.2)
Weight gain, n (%)	6 (19.4)
Cutaneous signs	
Scaling, n (%)	31 (100.0)
Erythema, n (%)	31 (100.0)
Hyperkeratosis, n (%)	12 (38.7)
Pustules, n (%)	2 (6.5)
Nail involvement	
Onycholysis, n (%)	20 (64.5)
Chronic paronychia, n (%)	16 (51.6)
Nail pitting, n (%)	14 (45.2)
Oil-drop discoloration spot, n (%)	11 (35.5)
Subungual hyperkeratosis, n (%)	6 (19.4)
Beau’s line, n (%)	1 (3.2)
Onychomadesis, n (%)	1 (3.2)
Mucosal involvement	
Conjunctivitis, n (%)	2 (6.5)
Oral mucositis, n (%)	2 (6.5)
Systemic and other signs	
Arthritis, n (%)	8 (25.8)
Fever, n (%)	5 (16.1)
Edema, n (%)	5 (16.1)
Lymphadenopathy, n (%)	2 (6.5)

Results from laboratory and diagnostic investigations are presented in Table 2. Hemoglobin and albumin values were, on average, 12.3 ± 2.0 g/dL and 3.8 ± 0.8 g/dL, respectively. The inflammatory profile showed an elevated mean erythrocyte sedimentation rate (ESR). A subset of patients demonstrated eosinophilia, and hepatic enzyme elevations were mild on average. Serologic testing identified hepatitis B surface antigen positivity in one patient (3.2%), whereas anti-hepatitis C virus and anti-HIV were negative in all tested patients (0%). Chest radiographs were normal in 30 out of 31 (96.8%) patients; one out of 31 (3.2%) demonstrated an abnormal finding. Systemic corticosteroids were the most commonly prescribed therapy, followed by methotrexate and acitretin. Azathioprine was used as a non-primary therapy, and adjunctive topical agents were selected at the treating clinician’s discretion. Overall, disease severity declined substantially from baseline by the third month and remained lower at subsequent follow-up visits. By the end of follow-up, a high proportion of patients had achieved a complete cure or clinical improvement, whereas a smaller subset had an unchanged disease status; worsening was uncommon. The distribution of outcome variables across time points is presented in Table 2.

**Table 2:** Diagnosis, investigations, management, and clinical outcomes of patients (n = 31) Abbreviations: DRESS, drug reaction with eosinophilia and systemic symptoms; BSA, body surface area; SD, standard deviation; IQR, interquartile range; Hb, hemoglobin; Hct, hematocrit; WBC, white blood cell; EOC, eosinophil count; ANC, absolute neutrophil count; AST, aspartate aminotransferase; ALT, alanine aminotransferase; BUN, blood urea nitrogen; ESR, erythrocyte sedimentation rate; HBsAg, hepatitis B surface antigen; HCV, hepatitis C virus; HIV, human immunodeficiency virus. Systemic therapy details: Methotrexate was administered at doses ranging from 7.5 to 12.5 mg per week (7.5 mg in three patients, 10 mg in two, and 12.5 mg in one). Acitretin was prescribed in eight patients, including seven who received 25 mg daily. Systemic corticosteroid regimens varied, including hydrocortisone 100 mg intravenously for two days, prednisolone 20 mg per day in several short-course treatments, and dexamethasone administered intravenously with tapering to oral prednisolone 30 mg, then 20 mg, and 15 mg per day, or dexamethasone 5 mg every eight hours followed by prednisolone 20 mg per day. Azathioprine was given at 50 mg daily in one patient, with unspecified doses in others. One patient received clotrimazole cream as adjunctive topical antifungal therapy. Data are presented as n (%) for categorical variables and as mean ± SD or median (IQR), as appropriate.

Variables	Overall (n = 31)
Diagnosis, n (%)	
Psoriasis	14 (45.2)
Eczema	8 (25.8)
Morbilliform drug allergy	5 (16.1)
DRESS	2 (6.5)
Fungal infection	1 (3.2)
Pemphigus foliaceus	1 (3.2)
Hematology and biochemistry, mean ± SD	
Hb (g/dL)	12.3 ± 2.0
Hct (%)	37.2 ± 5.9
WBC (per µL)	10,166.8 ± 3,492.0
EOC (per µL)	712.6 ± 650.6
ANC (per µL)	7,019.4 ± 3,669.6
Platelets (per µL)	290,516.1 ± 155,156.0
Total protein (g/dL)	7.1 ± 1.1
Albumin (g/dL)	3.8 ± 0.8
AST (U/L)	38.2 ± 18.6
ALT (U/L)	32.6 ± 30.6
BUN (mg/dL)	17.2 ± 11.7
Creatinine (mg/dL)	1.2 ± 1.0
ESR (mm/h)	28.7 ± 25.0
Serology and imaging, n (%)	
HBsAg positive	1 (3.2%)
Anti-HCV positive	0 (0.0%)
Anti-HIV positive	0 (0.0%)
Abnormal chest X-ray	1 (3.2%)
Systemic therapy, n (%)	
Methotrexate	6 (19.4)
Acitretin	8 (25.8)
Cyclosporin	1 (3.2)
Corticosteroid	14 (45.2)
Azathioprine	5 (16.1)
Topical/adjunctive treatment, n (%)	
Steroid	30 (96.8)
Vitamin D	11 (35.5)
Emollient	26 (83.9)
Other (clotrimazole cream)	1 (3.2)
Clinical follow-up	
Month 3	
Remaining BSA (%), median (IQR)	30 (45)
Outcome, n (%)	
Cured	6 (19.4)
Improved	25 (80.6)
Unchanged	0 (0.0)
Worsened	0 (0.0)
Month 6	
Remaining BSA (%), median (IQR)	10 (30)
Outcome, n (%)	
Cured	6 (19.4)
Improved	20 (64.5)
Unchanged	4 (12.9)
Worsened	1 (3.2)
Month 9	
Remaining BSA (%), median (IQR)	10 (20)
Outcome, n (%)	
Cured	8 (25.8)
Improved	15 (48.4)
Unchanged	4 (12.9)
Worsened	4 (12.9)
Month 12	
Remaining BSA (%), median (IQR)	3 (9)
Outcome, n (%)	
Cured	12 (38.7)
Improved	11 (35.5)
Unchanged	8 (25.8)
Worsened	0 (0.0)

The median percentage of body surface area involvement decreased from 100 (IQR, 5) at baseline to 30 (IQR, 45) at month 3, 10 (IQR, 30) at month 6, 10 (IQR, 20) at month 9, and 3 (IQR, 9) at month 12. Overall, BSA changed significantly over time (Friedman χ²(4) = 78.1, p < 0.001). In post-hoc analyses using Wilcoxon signed-rank tests with Bonferroni correction, body surface area at each follow-up visit (three, six, nine, and 12 months) was significantly lower than baseline (all adjusted p-values < 0.05). Throughout follow-up, outcome categories were distributed as follows: at month 3, six out of 31 (19.4%) were cured and 25/31 (80.6%) improved; at month 6, six out of 31 (19.4%) were cured, 20/31 (64.5%) improved, four out of 31 (12.9%) were unchanged, and one out of 31 (3.2%) worsened; at month 9, eight out of 31 (25.8%) were cured, 15 out of 31 (48.4%) improved, four out of 31 (12.9%) were unchanged, and four out of 31 (12.9%) worsened; and at month 12, 12 out of 31 (38.7%) were cured, 11 out of 31 (35.5%) improved, and eight out of 31 (25.8%) were unchanged. Exploratory within-person analyses did not identify statistically significant differences in the distribution of outcome categories across visits after multiplicity correction (Friedman test p = 0.371).

## Discussion

Given the heterogeneity in reported etiologies, clinical severity, and outcomes of ED across settings and the limited prospective evidence from Thailand, we conducted a prospective hospital-based cohort study to characterize the clinical presentation, causes, management, and outcomes of adult ED at a tertiary care center, with longitudinal follow-up for up to 12 months. We included 31 adults for analysis. Most were older men with multiple comorbidities who presented with generalized erythema, scaling, and pruritus involving a large body surface area. Pre-existing dermatoses, primarily psoriasis and eczema, were the most frequent underlying causes, followed by drug-related reactions. Most patients received systemic therapy, commonly systemic corticosteroids, acitretin, or methotrexate, alongside supportive care. Over follow-up, most patients achieved clinical improvement or a cure, and no deaths were observed.

The clinical and laboratory abnormalities in our cohort support the concept of ED as a final common pathway of immune dysregulation and profound barrier disruption. Previous studies have reported frequent laboratory abnormalities in ED, including anemia, leukocytosis, eosinophilia, and elevated ESR; these findings are biologically plausible in the context of T cell-mediated hypersensitivity in drug-induced disease [16] and autoreactive immune responses in autoimmune disorders such as bullous pemphigoid [17]. Generalized erythema and scaling are consistent with previous reports of marked impairment of the dermal barrier, predisposing to fluid, protein, and electrolyte loss and to metabolic and infectious complications [18]. These mechanisms provide a plausible explanation for the systemic manifestations we observed, even in the absence of significant clinical deterioration.

Our results regarding incidence, patient population, and etiology spectrum are consistent with those of previously published cohorts. A Thai study conducted at Sakon Nakhon Hospital documented an incidence of 160 cases per 100,000 dermatologic outpatients, a male-to-female ratio of 1.3:1, an average age of 50 years, psoriasis and eczema as the leading causes (53.1% and 31.3%, respectively), global pruritic scaly erythematous plaques, frequent alterations in nails, and fever [9]. In larger series, eczema (20.7%), psoriasis (16.8%), and drug-induced erythroderma (12.3%-46.6%) were frequently cited as etiologies, with male predominance and median age in the fifth to sixth decade [8, 19]. The greater predominance of psoriasis in our cohort aligns with data showing that erythrodermic psoriasis is an atypical but severe phenotype characterized by its occurrence in 1%-2.25% of patients with psoriasis and carries significant systemic risk [20]. These similarities strengthen the validity of our etiologic distribution and highlight the role of medications and chronic inflammatory dermatoses in adult ED.

Our findings are consistent with the prognostic gradient reported in previous studies, showing a favorable short-term course with no observed deaths and infrequent worsening during follow-up. This trend may reflect, in part, the etiologic profile in our cohort, in which inflammatory dermatoses and drug-related erythroderma were prominent, conditions generally associated with higher remission rates and lower mortality than lymphoproliferative etiologies [8, 9, 19]. In an Argentinian study involving 70 patients, 48.6% of the reasons for admission to the hospital were attributed to adverse drug reactions; in spite of common hypoproteinemia, elevated inflammatory markers, and eosinophilia, the in-hospital mortality attributed to erythroderma was only 3% [21]. In contrast, small cohorts of patients with AIDS and drug-associated erythroderma have had a significantly higher disease severity score and death rate of approximately 8% [22]. These findings highlight the potential role of underlying immunosuppression and comorbidities in influencing outcomes in erythroderma. Although malignancy-associated erythroderma is thought to be rare based on reviews reporting malignancy in 1%-3.3% of patients examined [23], in an elderly cohort in Singapore, where systematic screening occurred, malignancy was identified in 5.4% of cases and 7.8% in patients aged ≥ 65 years [24]. The absence of malignancy in our series should not be seen as an indication of negligible risk. Instead, it may be attributed to the small sample size, the relatively younger demographic of cases, and the absence of a standardized malignancy screening protocol. These findings underscore the importance of ongoing evaluation for underlying malignancy, especially in older patients or those presenting with atypical erythroderma. Although no patient in our cohort developed overt sepsis or organ failure or required intensive care, the nature of the systemic abnormalities identified the risks for serious complications of ED. Similar to burn injuries, damage to the epidermal barrier results in a high risk of developing bacterial and fungal infections, including bloodstream infection; contemporary data estimates the incidence of bloodstream infection at approximately 7.8% of cases of erythroderma and higher in people with abnormal temperature, chronic kidney disease, or hypoalbuminemia [25]. Opportunistic pathogens may further complicate the disease in immunocompromised patients [26]. Generalized skin involvement also results in the loss of fluids and electrolytes, contributes to hypoalbuminemia, and/or causes peripheral edema. There is also a risk of hemodynamic instability associated with a prolonged inflammatory response that affects cardiac function, leading to cardiac failure, hypothermia, or potentially multi-organ dysfunction if these clinical parameters are not addressed [27-30]. We suspected that the absence of serious infectious or organ-related events in our cohort is more reflective of early hospital admission, early supportive care, and predominantly inflammatory and drug-related causes for erythroderma rather than malignancy or defined immunosuppression. Nevertheless, these findings highlight the importance of close monitoring for possible infection, fluid balance, temperature, and organ function in every patient who presents with ED.

This study has several strengths, including its prospective cohort design, longitudinal follow-up for up to 12 months, and detailed clinical, laboratory, histopathologic, and treatment data, which provide pragmatic prospective evidence on adult ED in Thailand. We acknowledge several limitations. First, the study was conducted at a single academic care center, limiting the extrapolation of our results to patients managed in non-academic settings and non-community regions. Second, the sample size was small, which limited the statistical power and precision of estimates, especially for rarer causative conditions and infrequent, more severe complications. Third, referral patterns to a dermatology specialty clinic may have introduced referral bias, leading to overrepresentation of more complex or chronic cases compared with drug-induced or milder causes that may not have reached the clinic. Fourth, because etiologic classification and outcome assessment were based on routine clinical practice rather than a standardized investigative protocol, not all patients underwent the same screening for malignancy or advanced immunopathologic evaluation, which may have contributed to misclassification of some etiologic diagnoses. Finally, because treatment selection was based on clinician judgment rather than a standardized protocol, comparative evaluation of treatment regimens was limited. Future multicenter studies with more standardized diagnostic evaluation, malignancy screening, and treatment protocols are needed to improve etiologic attribution, identify prognostic factors, and better inform management across healthcare settings, including the role of biologic agents.

## Conclusions

In the single-center cohort reported here, adult ED mainly occurred in older patients with comorbidities and was prolonged, most commonly arising from pre-existing dermatoses (notably psoriasis and eczema), followed by drug-induced causes. Most patients required systemic therapy (corticosteroids, methotrexate, or acitretin) and supportive care, and most achieved at least some clinical improvement during follow-up, with no observed deaths and infrequent clinical worsening. This cohort reinforces ED as a final common pathway of diverse inflammatory and drug-related processes, highlights the value of early etiologic evaluation and monitoring for systemic complications, and supports larger multicenter or population-based studies to identify prognostic factors and optimize treatment strategies.
